# A Real-Time Fatigue Monitoring and Analysis System for Lower Extremity Muscles with Cycling Movement

**DOI:** 10.3390/s140712410

**Published:** 2014-07-10

**Authors:** Szi-Wen Chen, Jiunn-Woei Liaw, Hsiao-Lung Chan, Ya-Ju Chang, Chia-Hao Ku

**Affiliations:** 1 Department of Electronic Engineering, Chang Gung University, Taoyuan 333, Taiwan; E-Mails: chensw@mail.cgu.edu.tw (S.-W.C.); haoke.rev@gmail.com (C.-H.K.); 2 Healthy Aging Research Center (HARC), Chang Gung University, Taoyuan 333, Taiwan; 3 Department of Mechanical Engineering, Chang Gung University, Taoyuan 333, Taiwan; 4 Department of Electrical Engineering, Chang Gung University, Taoyuan 333, Taiwan; E-Mail: chanhl@mail.cgu.edu.tw; 5 Department of Physical Therapy and the Graduate Institute of Rehabilitation Science, College of Medicine, Chang Gung University, Taoyuan 333, Taiwan; E-Mail: yjchang@mail.cgu.edu.tw

**Keywords:** electromyogram, muscle fatigue, cycling movement, median frequency, fatigue progression measure

## Abstract

A real-time muscle fatigue monitoring system was developed to quantitatively detect the muscle fatigue of subjects during cycling movement, where a fatigue progression measure (FPM) was built-in. During the cycling movement, the electromyogram (EMG) signals of the vastus lateralis and gastrocnemius muscles in one leg as well as cycling speed are synchronously measured in a real-time fashion. In addition, the heart rate (HR) and the Borg rating of perceived exertion scale value are recorded per minute. Using the EMG signals, the electrical activity and median frequency (MF) are calculated per cycle. Moreover, the updated FPM, based on the percentage of reduced MF counts during cycling movement, is calculated to measure the onset time and the progressive process of muscle fatigue. To demonstrate the performance of our system, five young healthy subjects were recruited. Each subject was asked to maintain a fixed speed of 60 RPM, as best he/she could, under a constant load during the pedaling. When the speed reached 20 RPM or the HR reached the maximal training HR, the experiment was then terminated immediately. The experimental results show that the proposed system may provide an on-line fatigue monitoring and analysis for the lower extremity muscles during cycling movement.

## 1. Introduction

Some research has indicated that fatigue and decrease in muscle strength may serve as important indicators in frailty [1]. In fact, both factors are also considered as typical symptoms associated with patients with central nervous system damage [2–6]. For example, a previous study in the literature indicated that 50% of patients with Parkinson's disease (PD) showed symptoms of fatigue, even when a depressive mood disorder or cognitive impairment was excluded [7]. That is, fatigue has been proved to be a common and independent symptom in patients with PD without depression or dementia. On the other hand, it should be also noted that since fatigue is a negative symptom and not as obvious as other positive symptoms such as tremors, muscle stiffness or rigidity *etc.*, it is often ignored until severe motor function abnormality defects occur, therefore, the development of effective methods or mechanisms for detecting and assessing fatigue before severe degradation in motor functionality as well as devising effective anti-fatigue training methods are both very important.

In general, cycling based rhythmic contractions are similar to the control of walking since completion of these actions requires agonistic and antagonistic muscles to be alternately activated in coordination with a time sequence. For patients with poor coordination and balance control, a cycling ergometer bicycle can provide more adequate support for the trunk and help patients stretch their legs [8]. In addition, some researchers have also indicated that such movement is safe, effective and accessible to patients with a wide range of motor impairments [8–10]. Therefore, cycling-based movement is often used for walking training as well as the lower limb coordination training [11]. Previous research in literature even indicates that patients with stroke and cerebral palsy may improve their motor and balance abilities after an early short duration of cycling training [12,13].

Physical fatigue is normally accompanied by a progressive decline in motor function during motor tasks. In this aspect, a previous research report has proposed an experimental system to study physical fatigue during sustained and dynamic contractions [14]. On the other hand, it is indicated from another previous study that a prolonged cycling exercise would cause decrements in whole-body power, muscle-function, and jump-performance measures, implying that the exhaustion due to cycling exercise are related to fatigue in daily function [15]. Moreover, investigations into alternative measures for muscle fatigue as well as muscle activation during cycling were also conducted in previous research [16–18]. We may thus speculate that a system designed based on long-term repetitive exercise, such as the cycling-based movement, may be used as a tool for continuously, fully tracking and assessing fatigue or degeneration of motor functional activities. Therefore, the aim of this study is to develop a system for producing and quantitatively analyzing the kinesiological and kinematical data to assess the fatigue during cycling movement at a constant workload.

In this paper, a new cycling training system for on-line continuous monitoring and analysis of fatigue-related parameters is introduced. In order to achieve the goal of real-time muscle fatigue assessment, the EMG activities of dominant muscle groups are first recorded for kinesiological analysis during a cycling-based motor task. We also propose an innovative and new-defined indicator, dubbed fatigue progression measure (FPM), to quantitatively characterize the fatigue. It is worth noting that the onset time of the occurrence of fatigue can be explicitly determined from the FPM traces. This would represent one of the most significant benefits from our research. In the authors' opinion, the proposed system can not only provide an on-line fatigue monitoring and analysis, but also represent a benefit to clinical practitioners for assessing the cycling exercise-based training progress. Also, the innovative system may further effectively applied as an anti-fatigue training device in a variety of physical therapy or rehabilitation-related fields.

## 2. System Configuration

### 2.1. System Features

**Figure 1.**
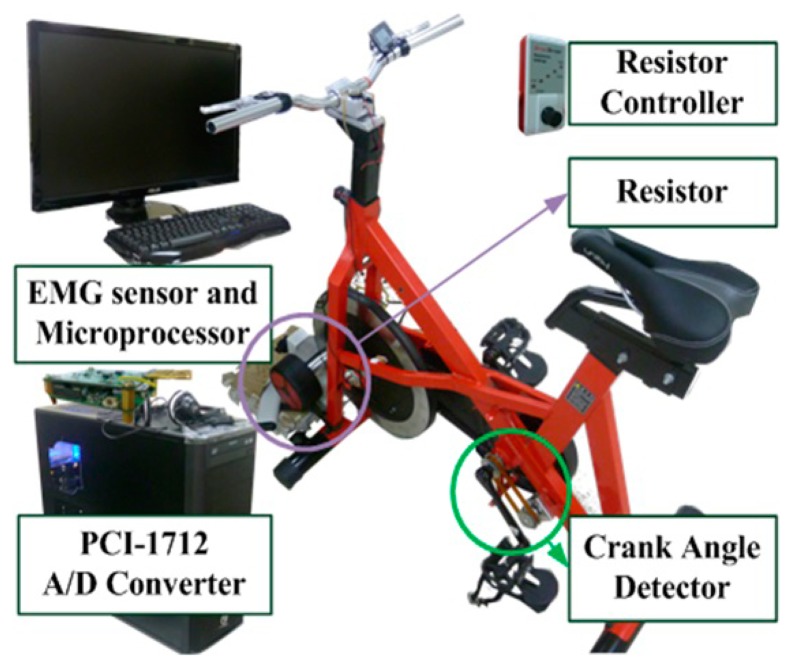
System setup for the proposed real-time fatigue monitoring and analysis for lower extremity muscle. The cycling-based system is consisting of a physical bicycle equipped with a resistor, crank angle detector and the wireless 2-channel EMG sensors with sensor interface device.

In general, the overall system is devised for detecting and assessing fatigue during cycling in a real-time fashion. The system consists of a physical bicycle equipped with a number of peripheral components/devices, a set of wireless EMG sensors with sensor interface device, and a computational unit in which software for providing visual feedback as well as processing the on-line measured EMG signals is implemented, as shown in Figure 1. The computational unit comprises a computer system (Core i7, Windows 7), a LabView-based software program for providing the on-line monitoring of the cycling speed as well as the EMG analyzing results during the cycling, a high-performance Graphic Processing Unit (GPU) for hardware acceleration (NVIDIA GeForce GTX 560 Ti), and an Analog-to-Digital Converter (ADC, 16 ch, 12 bit, PCI-1712).

The bicycle in our system allows subjects to perform cycling training exercise simply by stepping on the pedals. When cycling starts, the wearable wireless sensors with sensor interface device synchronously collect EMG data for real-time fatigue analysis during the test. In addition, other factors such as the cycling speed and orientation angles may also be displayed in a real-time manner so a visual feedback can be provided for the subject during the cycling process. The computer monitor (28 inches) is used as a display device that shows the fatigue analysis results and visual feedback.

### 2.2. Cycling Position and Velocity Detection

**Figure 2.**
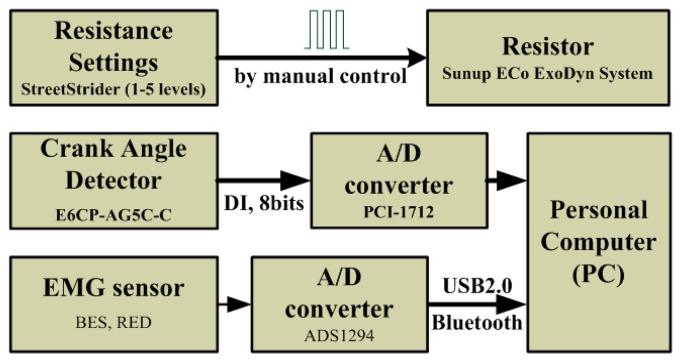
Schematic block diagram of overall system configuration.

As described previously, a physical bicycle equipped with an electromagnetic load was modified to fit in the proposed system and serve as a cycling platform. The cycling speed is determined at the time when the subject is stepping on the bicycle pedals. In order to estimate the position and velocity, an optical encoder (OMRON E6CP-AG5C-C) with a resolution of 256 pulses/cycle was employed to detect the absolute crank position in degree (from 0 to 360) for kinematical analysis. According to the schematic block diagram as shown in Figure 2, in order to detect the cycling or crank position, the digital output of the encoder is transmitted into the computer in the form of Gray code through the data acquisition board (PCI-1712) so the cycling position may be determined in the virtual environment provided by the computer. Moreover, since the value of cycling position is obtained, the cycling angular velocity can be computed, thus the rotation speed of the bicycle can be then determined.

### 2.3. Load Control Device

A load control device was utilized to provide an amount of workload imposed to the cycling-based exercise. The device is consisting of a load controller and workload generator, as illustrated in Figure 1. The load controller was used to control the size of electromagnetic load using a pulse width modulation (PWM) signal. The workload generator (*i.e.*, a bicycle trainer), which has a similar function to a brake, was employed for providing the load to the bicycle.

### 2.4. Electromyography (EMG) Sensing Device

In our study, we adopted an EMG sensor designed and built by CGU BES lab. The sensor is consisting of 2 channels with 1 KHz sampling rate. The device collects and transmits the EMG data to PC using Bluetooth protocol without data loss.

### 2.5. Software Settings

**Figure 3.**
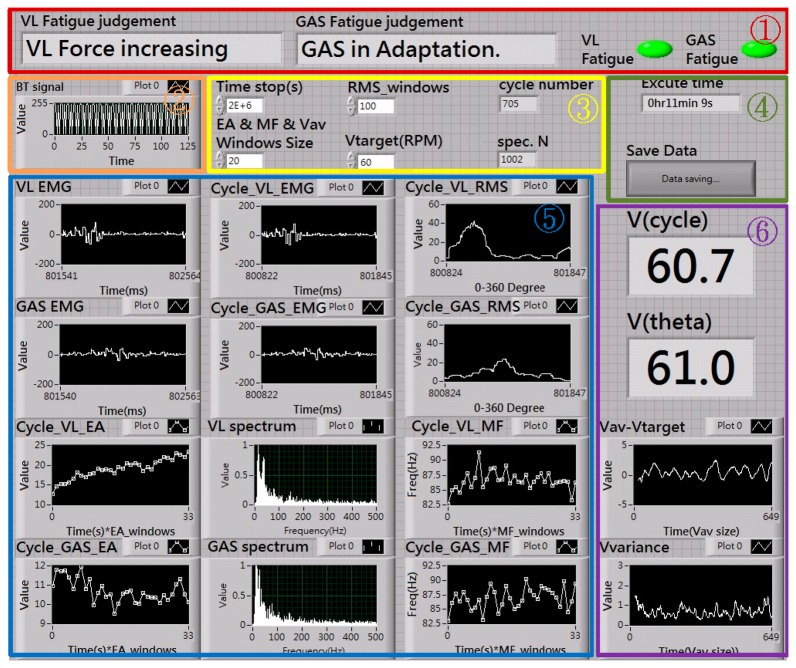
Display of kinematical data and analysis using LabView windows. In these windows, cycling speed, EMG signals, MFs and EAs of both the VL and GAS muscles for each cycle are shown.

In software settings, most of the system software was developed and implemented in the LabView environment. These software programs were established to acquire data, assess and detect the fatigue in an integrated fashion using the LabView version 2010 for Microsoft Windows 7. In general, the software was devised to carry out the following functions/tasks in a real-time manner: (1) acquiring and displaying the EMG data and cycling speed transmitted from the sensors in the system, (2) preprocessing the collected raw EMG data, (3) performing fast Fourier transform (FFT) based power spectral calculations of the EMG signals per cycle, (4) integrating the EMG spectral analysis results into force- and fatigue-related parameters (*i.e.*, EA and MF as indicated in Section 3.3) used for on-line fatigue detection. Figure 3 shows the display panels of the kinematical data and kinesiological analysis in our system implemented using LabView windows.

## 3. Methods and Materials

### 3.1. Subjects and Settings

In our experiment, five healthy adults were recruited for performing the bipedal cycling training so both the kinesiological and kinematical data required for the subsequent investigations into fatigue detection can be produced. All the subjects were free of any muscular or neurological disorders. Their mean age was 23 years old and they all had at least 10 years of cycling experience. Before proceeding, we explained to them the procedures of the experiment and the characteristics of the bicycle ergometer system. All subjects were asked to sit in an upright position and maintain a constant speed by referring to the visual feedback information of their current velocities as best as they could. During the pedaling process, EMG of VL and GAS muscles, crank position, instantaneous cycling velocity and cycling time were recorded. Also note that here we hypothesized that once fatigue occurs, the cycling speed would show larger deviations from the target speed, and thus a measure of deviation in velocity was also defined and used as a reference for judging the stability of cycling. The study protocol had been approved by Chang Gung Medical Foundation Institutional Review Board (IRB no.101-5141B) in accordance with the Helsinki Declaration. All participants have provided their written consent, approved by Chang Gung Medical Foundation Institutional Review Board (IRB no.101-5141B), to participate in this study.

### 3.2. Experimental Procedure

Prior to experiment, each subject was asked to do one-minute low intensity exercise to warm up body to prevent sports injuries. Then, the experiment began and each subject performed cycling exercise under a constant load throughout the course of experiment. During the cycling exercise, all the subjects were asked to pedal at a steady cadence and maintain a constant speed of 60 RPM. Note that the experiment was performed on the basis of the visual feedback in which the subject's velocity is available in real time on the screen. In addition, since the Borg rating of perceived exertion (RPE) scale is known as a useful way to reflect how hard a subject feels the work is while he/she is exercising [19], in our study both the heart rate (*HR*) and Borg scale value were also simultaneously recorded and updated every minute during the test.

Further note that here a method, referred to as the Karvonen method, was adopted for determining the training *HR* range in our study. In general, it is a mathematical formula that involves adding a given percentage of the maximal heart rate reserve (*i.e.*, the maximal heart rate subtracting the resting heart rate) to the resting heart rate, as expressed by:

(1) $$HR_{\text{target}}=(HR_{\max}‐HR_{\text{rest}})\times EI+HR_{\text{rest}}$$

where *HR*_target_, *HR*_max_, *HR*_rest_, represent the target, maximal, and resting heart rate, respectively; *EI* denotes the exercise intensity in percentage. In our study, we set *EI* to 60%–80%. Staying within this range of *HR* will help one work most effectively during the subject's cardio workouts. Once the Borg RPE scale value of a subject reached the maximal value of 20 or the subject's *HR* reached the maximal training *HR*, the experiment was then terminated immediately.

### 3.3. Kinesiological Data Analysis

In this study, surface EMG signals measured from various muscles were digitized and collected using a wirelessly telemetric procedure. As mentioned previously, the sampling rate was set to 1 KHz. The raw EMG segment derived from each cycle (*i.e.*, from 0 to 360 degrees in cycling) was preprocessed by subtracting the mean, *i.e.*, the DC term, as well as bandpass filtering. Then, both the electrical activity (EA) and the median frequency (MF) were evaluated on the raw EMG segment. For this purpose, a moving Root-Mean-Squared (RMS) based envelope detection was performed over a time window of 100 ms by shifting the window by a step of 1 ms throughout the entire cycle. Then, the EA was evaluated by integrating the RMS curve (*i.e.*, calculating the area under the envelope) over the cycle, as expressed in:

(2) $$EA=\frac{1}{N}\sum_{i=1}^{N}\text{RMS}(i)$$

where RMS(*i*) denotes the discrete-time RMS value and N denotes the total number of RMS values within a cycle. It should be noted that EA can be viewed as a force-related measure.

On the other hand, the EMG spectra were also simultaneously calculated from the raw EMG segment over a cycle by Fast Fourier Transforms (FFT), and the Median Frequency (MF) corresponding to the cycle was then determined by:

(3) $$\int_{0}^{MF}\text{SPEC}(f)df=\frac{1}{2}\int_{0}^{\infty }\text{SPEC}(f)df$$

where *SPEC*(*f*) represents the power spectral density (PSD) of raw EMG segment measured from a cycle.

In fact, the reason that we computed EA and MF is it is indicated by a previous research called Joint Analysis of EMG Spectrum and Amplitude (JASA) that the fatigue indication would be more reliable if both the changes in the amplitude and the spectrum were considered simultaneously [20,21]. The EMG amplitude is quantified by EA as given by Equation (2) while the spectral information is characterized by MF as given by Equation (3). In general, EA is a force-related parameter while MF is a fatigue-induced parameter.

It should be noted that when applying the JASA methodology, knowledge about the temporal change in both the EMG amplitude and spectrum is needed. Therefore, in our system both the EA and MF estimates are evaluated and updated every minute as long as a subject's HR stays within the target training range of HR as indicated in Equation (1).

### 3.4. An Innovative New-Defined Indicator of Muscle Fatigue—FPM

As indicated by previous researches [20,21], it is well known that a decrease in MF is an indication of muscle fatigue. In this study, we devised an innovative and novel parameter for quantitatively measuring the degree of fatigue in real time. The dubbed FPM is defined as:

(4) $$\text{FPM}=\frac{\text{Number of events with MF lower than the reference}}{\text{Total number of events}}$$

Equation (4) is an accumulated fractional function, where the numerator is the number of the events of MF lower than the reference value and the denominator is the total number of the events counted. Denoting the first MF obtained from the cycling test as *MF*_1_, the reference value is simply set to (*MF*_1_ − *f*_nm_) Hz, where *f*_nm_ represents a quantity referred to as the noise margin. In fact, in electrical engineering, noise margin is the minimum amount by which a signal exceeds the threshold for proper operation. In order to minimize the number of false alarms due to noise-corrupted MF values, we here incorporated the noise margin into the FPM algorithm for the first time. In our study, we set *f*_nm_ = 0.5.

In fact, it should be noted that obtaining a new solution to dynamically and continuously monitoring the progression of muscle fatigue in a more practical situation is desired and crucial in our work. In this aspect, our FPM, by definition, can effectively convert the EMG-related fatigue measure MF into diagnostically useful time-domain parameter, thus being able to quantitatively describe the occurrence as well as the progression of muscle fatigue as time evolves.

**Figure 4.**
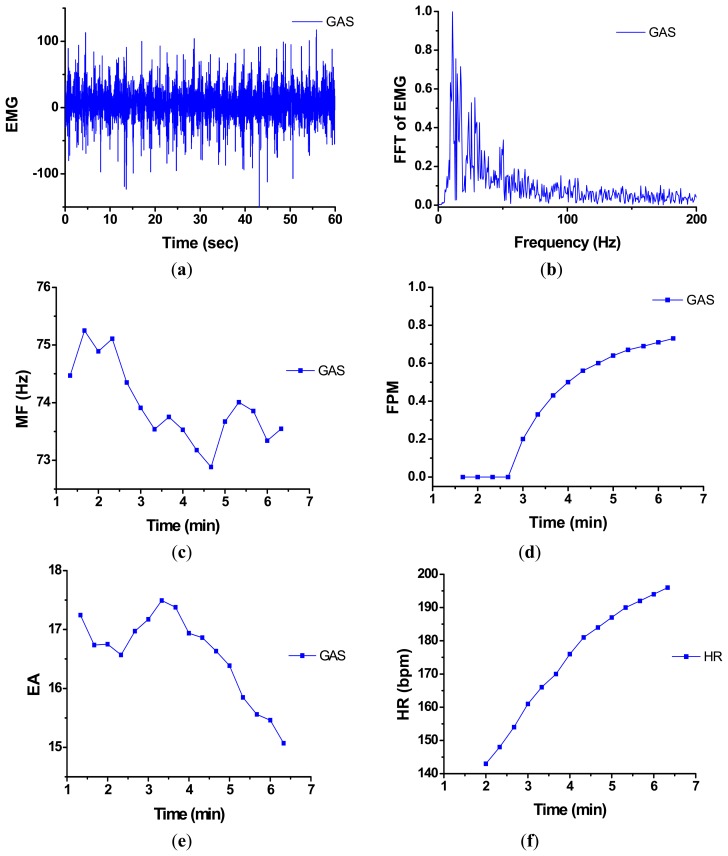
Real-time profiling of a typical test obtained from the proposed system: (**a**) raw EMG; (**b**) FFT-based spectrum of the raw EMG signal; (**c**) MF estimates derived from the EMG; (**d**) FPM tracings; (**e**) EA estimates derived from the EMG; and (**f**) HR measurements.

According to the descriptions of Section 3.3, each time there was an MF obtained from the spectrum of an EMG segment measured over one cycle (1 s) during pedaling. Denoting the MF sequence as *x*(*n*), the sequence was then passed into a moving average filter (MAF). In our study, the input-output relation of the MAF process is characterized as:

(5) $$y(n)=\frac{1}{M}\sum_{k=1}^{M}x(n\cdot S+M-k)$$

where *y*(*n*) represents the output data (*n* = 0,1,2,…), *M* is called the filter length or alternatively known as the averaging window length, and S is the shift of the moving window. In general, it is indicated from Equation (5) that the output *y*(*n*) is actually obtained by taking an average over an *M*-point windowed MF segment, then shifting the *M*-point window by step of *S* and redoing the same *M*-point averaging process throughout the entire input MF sequence. In fact, the overall system as indicated in Equation (5) is a linear lowpass filtering (LPF) process. Such a linear filtering process can make the MF sequence smoother so the subsequent analysis might be easier to be manipulated. Note that here we set *M* = 60 (*i.e.*, one minute in real time) and *S* = 20 (*i.e.*, 20 s in real time). As a result, the filtered MF value was produced every 20 s (*i.e.*, 1/3 min) and thus, the total number of events counted increases by one per 1/3 min. Therefore, the FPM, as defined in Equation (4), is a time-varying parameter that can be used for on-line monitoring and updating the progressive fatigue in every 20 s. The real-time profile of a typical test, consisting of a raw EMG signal, the corresponding FFT-based spectrum, MF, FPM, EA, and HR, is demonstrated in Figure 4.

Figure 4a shows a 1-minute EMG segment recorded during the cycling exercise. It is actually revealed from Figure 4a that the subject was cycling at a speed of about 60 RPM and the highest EMG amplitudes should occur during the pedaling periods. Then, FFT-based PSD of the raw EMG was calculated, as depicted in Figure 4b. After that, the MF can be found from the PSD. Repeating the above two steps by shifting a 1-minute analysis window by a step of 20 s on the input EMG data, we may then obtain the running estimate of MF signal in a real-time manner, as shown in Figure 4c. Observing Figure 4c, a decrease in MF can be actually regarded as the result of muscle fatigue (GAS). The FPM tracings corresponding to the MF signal was further evaluated in the following steps. First, as described previously, the threshold involved in FPM computation is set to (*MF*_1_ − 0.5) Hz. Since it is revealed from Figure 4c that *MF*_1_ ≈ 74.5 Hz, we set the threshold to 74 Hz. Next, the FPM was then evaluated by successively comparing the subsequent MF values with the reference value, 74, using Equation (4). As a result, the FPM tracings corresponding to the MF signal was finally obtained and plotted in Figure 4d. According to Figure 4d, we may see that FPM can not only provide a quantitative description of muscle fatigue progression, but also detect the onset of muscle fatigue. Moreover, Figures 4e and 4f show the real-time EA estimates and HR measurements, respectively, during the cycling process.

## 4. Results and Discussion

In order to evaluate the performance of the proposed system in actual practice, we built up the system and then used it to collect the raw EMG data derived from VL and GAS muscles of the five healthy subjects during the bipedal cycling movement. Kinesiological analysis such as FFT-based PSD, EA and MF calculations were all synchronously performed and displayed by the system software. In addition, FPM tracings were also generated. All these results were jointly applied for on-line fatigue assessment. It should be noted that among all these EMG-related factors, FPM method is an innovative and new technology. In fact, it can not only characterize the progression of fatigue, but also determine the explicit onset time of the fatigue occurrence, thus representing the most significant benefit from our research. Therefore, the subsequent outcome analysis and discussion will be more focused on the performance evaluation of FPM method.

### 4.1. Outcome Analysis

Note that in our study, different loads were applied for each individual subject, according to their muscular strength and endurance. Evaluation on FPM stopped at the time when the subject's HR achieved the maximum (*i.e., EI* = 80%) of his/her training HR during the test or Borg RPE scale value reached 20 [22].

**Figure 5.**
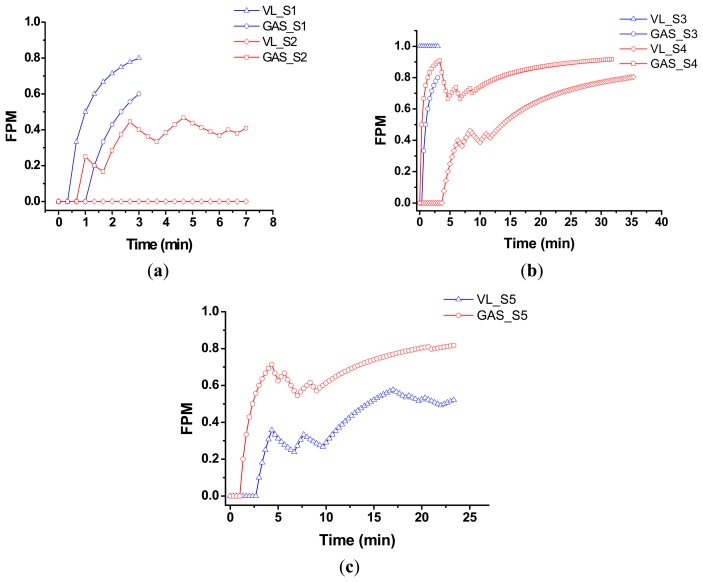
FPMs of VL and GAS muscles *versus* time for (**a**) S1 and S2 under L2 load; (**b**) S3 and S4 under L3 load; and (**c**) S5 under L4 load.

Figure 5 shows typical FPM-*versus*-time plots of five subjects' GAS and VL evaluated right after one-minute warm up. Figure 5a, b and c provides the results with different loads, where L2, L3 and L4 denote the light, middle and heavy loads, respectively. For example, L2 was assigned for two females with light physical activity (subjects S1, S2), and L4 for an athletic male (subject S5). In addition, L3 was for two males (subjects S3, S4), where S4 is with moderate physical activity and S3 with light activity. Figure 5 indicates that the FPMs of GAS and VL of these subjects are significantly different. Under light load (L2), the fatigue of GAS leads to that of VL for S1, whereas there is no sign of fatigue for S2's GAS. For S3 under middle load, GAS exhibits completely fatigued at the beginning, and his VL gradually becomes fatigued. Finally, the test of S3 was terminated after 3 min due to his Borg RPE reached 20. Of interest is that the *EI* of HRs of S3 and S5 were only 60% when their Borg RPE reached 20. In contrast, the HRs of S1, S2 and S4 were the 80% of their training HRs when their RPE reached 20. It implies that Borg RPE is a combination of HR and muscle fatigue. For S4 with L3 and S5 with L4, their GAS got fatigued earlier than VL. Moreover, the fluctuations in the progressive developments of both GAS's and VL's fatigue for S2, S4 and S5 were also observed. This could be attributed to that these subjects adjusted and changed their postures and attitudes during the cycling motion in order to reduce muscular fatigue [23]. The coordination and compensation of muscle group may reduce the local muscle fatigue, and exert the same output force to perform the constant-speed cycling under a constant load.

### 4.2. Discussion

First, observing Figure 5, we may find that the FPM evolutions of these typical cases seem to increase exponentially with time at the beginning, and thus we modeled it simply using a continuous-time exponential function as:

(6) $$\text{FPM}(t)=1-e^{-(t-t_{\text{on}})/T}$$

where *t*_on_ represents the onset time of the occurrence of muscle fatigue; *T* is referred to as the Time Constant (TC), a quantity used for estimating the time required for the FPM curve to reach approximately 63.2% of the maximum level (of fatigue). The onset times of GAS and VL of these five subjects under different loads are listed in Table 1. Note that Equation (6) is an approximation, where *T* can be actually regarded as a quantity presenting the time required for a subject to become completely fatigue after the onset of fatigue. Alternatively, *FPM*(*t*) can be further expressed as:

(7) $$\text{FPM}(t)=1-e^{-k(t-t_{\text{on}})}$$

where *k* = 1/*T*. In fact, *k* is a novel fatigue-induced parameter introduced by our study; we called it the “fatigue incremental rate” (FIR). Furthermore, we can derive and obtain *T* ≅ *t*_on_, if a subject's muscle continues to exhibit fatigue after the onset time *t*_on_. The detailed derivation is provided in Appendix A. We speculate that these model parameters *t*_on_, *k*, and *T* may show the subject's performance of muscle endurance. Hence, our model of muscle fatigue assessment may allow effective and wide applicability in the physical therapy or rehabilitation-related fields involving cycling training.

Further inspecting results presented in Figure 5b–c, one may see an exponential increase in FPM generally followed by a drop. As indicated previously in Section 4.1, this could be attributed to that these subjects (S4 and S5) started to adjust and change their postures and attitudes after fatigue occurred, in order to reduce muscular fatigue during the cycling motion. Since they adaptively regulate muscle groups for reducing local muscular fatigue, a decrease in FPM following an exponential increase in FPM was then observed. Meanwhile, when reducing the local muscular fatigue by spontaneously coordinating and compensating muscle groups, the subject also exerted the same output force to perform the constant-speed cycling under a constant load. This might effectively defer the progressive fatigue of a specific muscle, thus resulting in a different relatively slower exponential increase profile (*i.e.*, a smaller FIR value) after a drop in FPM.

Moreover, as mentioned previously, since the Borg scale can be used to quantitatively monitor the intensity of exercise [19], each subject was also asked to rate the degree of perceived exertion he/her felt during pedaling. According to Borg scale, subjects may rate their perception of the exertion 13 on scale when they feel the exercise intensity is “somewhat hard”. We speculate that this scale value can reflect the physical fatigue and thus may be used to validate the FPM method. Therefore, we here also included the occurrence times of these five subjects' rating 13 on Borg scale during the cycling exercise in Table 1. Inspecting and comparing all the numerical results of onset times as listed in Table 1, we may see that for each subject the onset time of muscle fatigue, either due to GAS or VL, and that of Borg = 13 were close to each other, suggesting that the results obtained from different ways of fatigue detection were actually consistent to each other. Also, it is revealed from Table 1 that the physical fatigue seemed to occur shortly prior to the perception of fatigue (within 1 min or less).

Furthermore, it is revealed from Table 1 that muscle fatigue occurred at 2 min 40 s for S5, yet he still cycled for 23 min. The difference between these two times is explained as follows. First, it should be noted that here 2 min 40 s actually was just the onset time at which muscle fatigue occurred for the first time during the cycling motion test. Secondly, since different subjects should have different muscular strengths and endurances, subject S5, for sure, should have better muscular strength and endurance than the other subjects. That is why he was still able to continuously pedal for 23 min, even though his muscle started to fatigue at 2 min 40 s. In addition, another possible reason could be due to that different subjects might use different strategies in coordinating and compensating their muscle groups to reduce the local muscle fatigue so they may be able to have longer endurance times during the test. Therefore, we may speculate that subject S5 should not only have a better muscular strength and endurance, but also might have a better strategy to perform the task of the cycling motion test. Since the Borg scale value reflects the individual subjective sense, a mixing of the mental fatigue and peripheral one, the onset time of the muscle fatigue estimated from FPM is not necessarily in accordance with the outcome performance (the total time of pedaling) of each person.

Finally, it could be useful to know how the FPM would recover when a subject is no longer in a fatigue state. According to the FPM model as indicated in Equation (4), one may see that the longer a subject is in fatigue, the larger the numerator of FPM is. This will cause more time for FPM to decay to zero once the subject is no longer in a fatigue state. That is, it would take more time to recover from fatigue. Therefore, we may conclude that when a subject is no longer in a fatigue state, the time required for the person to recover from fatigue basically depends on how long he/she was in fatigue.

## 5. Conclusions

A cycling-based system for real-time muscle fatigue monitoring and assessment was developed in this study. In this integrated system, the kinesiological and kinematical data, including the EMG signals of the VL and GAS muscles in one leg, cycling speed and crank angle are synchronously measured. In addition, the EA and MF of EMG signals are calculated. Moreover, a novel FPM, which is the percentage of the reduced MF counts, was proposed and built in this system to on-line and continuously quantify the status of muscle fatigue as time evolves in pedaling exercise. Five young healthy subjects were recruited to participate in the cycling experiment. Our results demonstrate that this method can be successfully applied on a bicycle ergometer for the real-time characterization and monitoring of the onset and the progression of lower extremity muscles' fatigue. Our study actually provides a paradigm that has guiding meaning of methodology for continuously tracking and quantitatively characterizing the fatigue progression during motor tasks. In summary, our study has demonstrated the feasibility of using this method for on-line monitoring, diagnosis or assessment of muscular fatigue to avoid an over-exercise. In the future, it could be further applied to the related studies regarding the muscle training, physical therapy or rehabilitation fields.

## Figures and Tables

**Table 1.** Onset times of muscle fatigue (both GAS and VL) and Borg = 13 (indicating that the exercise intensity is “somewhat hard”) of the subjects under different loads. Note that for each subject the Borg scale value was updated every minute during the test.

**Load**	**Subject**	**Gender**	**Fatigue Onset (GAS)**	**Fatigue Onset (VL)**	**Onset of Borg = 13**	**Total Time of Pedaling**
L2	S1	Female	20 s	1 min	2 min	3 min
	S2	Female	0	40 s	2 min	7 min

L3	S3	Male	0	20 s	1 min	2 min 40 s
	S4	Male	3 min 40 s	0	4 min	36 min

L4	S5	Male	2 min 40 s	1 min	3 min	23 min

## Appendix A

If a subject's muscle continues to exhibit fatigue after the onset time ton, according to Equation (4) a discrete-time version of FPM can be expressed as:

(A1) $$\text{FPM}(i)=\frac{i}{n_{\text{on}}+i},i\geq 0$$

where *n*_on_ is the number of events before the onset, and *i* denotes the *i*-th event after the onset. At the beginning of fatigue onset, *i* is smaller than *n*, and then the fractional function, according to the binomial theorem, can be approximated as:

(A2) $$\text{FPM}(i)≅\frac{i}{n_{\text{on}}}\left[1-\left(\frac{i}{n_{\text{on}}}\right)\right]$$

On the other hand, the exponential function as indicated in Equation (6) can be approximated by Taylor's expansion as:

(A3) $$\text{FPM}(i)≅\frac{i\Delta t}{T}+\frac{1}{2}{\left(\frac{i\Delta t}{T}\right)}^{2}$$

Where Δ*t* is the time interval of event, where Δ*t* = 20 s. From the comparison of the leading behavior of Equations (A2) and (A3), we can obtain the relation, *T* ≅ *n*_on_ Δ*t* = *t*_on_.
